# The healing power of music: a mixed-methods study on stress reduction in paediatric hospitalisation

**DOI:** 10.1186/s12906-025-05098-0

**Published:** 2025-10-17

**Authors:** Ivone Nunes da Silva Santa, Maria Lucia Barbosa Maia dos Santos, Lucca Garcia Moreira Ribeiro, Danton Matheus de Souza, Ana Paula Scoleze Ferrer

**Affiliations:** 1 Specialist in Therapeutic Education and Social Therapy, University Hospital, University of São Paulo. São Paulo (USP), São Paulo, SP Brazil; 2 Department of Pediatrics, School of Medicine, University of São Paulo, São Paulo, SP Brazil; 3 School of Nursing, University of São Paulo (USP), São Paulo, Brazil SP; 4 School of Nursing, Federal University of São Paulo (UNIFESP), São Paulo, SP Brazil; 5 Department of Pediatrics, School of Medicine, University of São Paulo, Avenida Dr Eneas de Carvalho Aguiar, 647, São Paulo, CEP: 05403-000 SP Brazil

**Keywords:** Music-based interventions, Complementary and integrative health practices, Humanised care, Paediatric hospital care

## Abstract

**Background and objectives:**

Hospitalisation is a potentially distressing experience for children and their families, often accompanied by emotional, psychological, and physical discomfort. Humanised care through complementary and integrative health practices (CIHP), such as music-based interventions (MBIs), has demonstrated beneficial effects in specific paediatric populations. However, evidence in general paediatric hospital settings remains limited. This study aimed to assess the impact of an MBI on perceived stress and the subjective experience of hospitalisation among paediatric patients and their caregivers.

**Methods:**

It was a quasi-experimental study, cross-sectional, with a mixed-methods approach, conducted in a paediatric ward of a teaching hospital affiliated with the University of São Paulo. The study carried out in two phases: (I) a quantitative evaluation of perceived stress using a visual analogue scale (VAS) before and after the intervention; and (II) a qualitative analysis of caregivers’ perceptions through semi-structured interviews. The intervention included 20–40 min of live music using string instruments (kantele or lyre), complemented by age-appropriate singing and spoken word. Quantitative data were analysed using the Wilcoxon test; qualitative data underwent content analysis and lexical analysis via IRAMUTEQ software.

**Results:**

A total of 125 children (mean age: 30.6 months) and their caregivers participated in Phase I. On a scale from 0 to 10, the mean stress score decreased by 2.18 points among children/adolescents and by 1.45 points among caregivers, with the reduction being significant for both (*p* < 0.001), with a large effect size (Cohen’s d = 1.0). All participants approved the intervention, and 74.4% reported behavioural improvements in the children. Phase II included 30 interviews, which revealed overwhelmingly positive perceptions of the intervention. Caregivers described the hospital environment as stressful and isolating but reported that the MBI provided relaxation, comfort, and emotional relief for both children and themselves.

**Conclusions:**

The findings highlight the potential of MBIs as an effective, safe and non-pharmacological strategy to reduce stress and improve emotional well-being for paediatric patients and their families. Incorporating music-based practices into routine paediatric care may foster more welcoming, humanised environments that address the emotional needs of children and caregivers.

**Supplementary Information:**

The online version contains supplementary material available at 10.1186/s12906-025-05098-0.

## Background

Hospitalisation is a potentially stressful event, often associated with physical, emotional, and psychological distress for patients and their families. The hospital environment is unfamiliar, frequently perceived as hostile, and characterised by invasive and painful procedures, which distance individuals from their usual activities. These aspects are typically accompanied by feelings of fear, anxiety, sadness, and worry [1–3]. A population-based study involving more than 280,000 patients aged between 3 and 21 years revealed that 7% received a diagnosis of mental health problems following hospitalisation [4]. This prevalence is likely to be underestimated, as the majority of psychological problems may not receive a formal diagnosis and therefore are not accounted for.

In response to this scenario, several strategies have been proposed to mitigate the impacts of hospitalisation on children and their caregivers. Numerous humanised initiatives have shown positive results in improving the experience of patients, families, and healthcare professionals when holistic care approaches are incorporated. Humanised care contributes to alleviating psychological distress and enhancing adherence to therapeutic proposals, thereby improving treatment effectiveness [5–7]. Complementary and Integrative Health Practices (CIHP) have increasingly been integrated as key tools for humanised care, offering complementary forms of support aimed at promoting body–mind balance, reducing stress, and improving quality of life [8, 9]. These strategies are safe and low-cost, and are particularly important in settings with limited psychological support resources within the hospital environment.

Art-based practices have been increasingly utilised as strategies for humanising healthcare. Studies have demonstrated beneficial effects of art-based therapies across various clinical conditions, aiding in emotional regulation, improving quality of life, and reducing symptoms of anxiety, depression, and pain [10]. Music-based interventions (MBIs), as a form of integrative practice, have been widely used to promote health and well-being, owing to their strong emotional appeal. MBIs encompass various ways of using music for therapeutic purposes within healthcare, which can be categorised into three main types: music medicine refers to the use of recorded music, provided by healthcare professionals, to reduce stress and promote well-being before, during or after medical procedures; music therapy, in turn, involves structured interventions delivered exclusively by qualified and accredited music therapists, with clearly defined therapeutic objectives, and finally, other MBIs include musical activities carried out by musicians or healthcare professionals without formal training in music therapy, with recreational, educational or health-promotion purposes [11].

The first report of music therapy in children and adolescents’ dates to the late 1950 s, when it began to be used as a therapeutic tool for mental health conditions (Davis, W.B., cited by Stegemann et al. [11]). Since then, it has demonstrated positive clinical outcomes in various conditions, such as epilepsy, autism spectrum disorder, depression, and other mental health disorders, as well as in the rehabilitation of children with disabilities and neurological impairments [11–13]. In hospital settings, MBIs have been used as non-pharmacological interventions to address pain, alleviate stress and anxiety, and promote well-being, particularly among oncology patients, those in palliative care, and neonates in intensive care units [11, 14, 15].

Despite the growing recognition of the benefits of MBIs in promoting health and well-being, and substantial evidence supporting the benefits of MBIs for hospitalised children, paediatric populations remain underrepresented in research on this topic. Most of the existing evidence focuses on adults, particularly in contexts such as chronic pain, mental health, or palliative care. Among children, studies are more frequently conducted in specific hospital settings, such as oncology, neonatal patients or intensive care, while the use of MBIs in general paediatric care remains scarce. Few studies have been conducted in paediatric wards, especially using a mixed-methods approach in the context of low- and middle-income countries. This gap may be attributed to methodological factors, including ethical in conducting research with children, as well as the mistaken perception that the benefits of music are automatic or universal, which reduces the incentive for rigorous investigation of its effects. Although recognised by the World Health Organization (WHO), CIHP remain limited in paediatrics, and the American Academy of Pediatrics (AAP) highlights the need for reliable and scientifically grounded information in this field [16].

Accordingly, this study aimed to evaluate the effects of an MBI as a complementary and humanised care resource for hospitalised paediatric patients and their caregivers. The outcomes assessed included self-perceived stress and meanings attributed to the hospitalisation experience before and after the proposed intervention.

## Methods

**Fig. 1:**
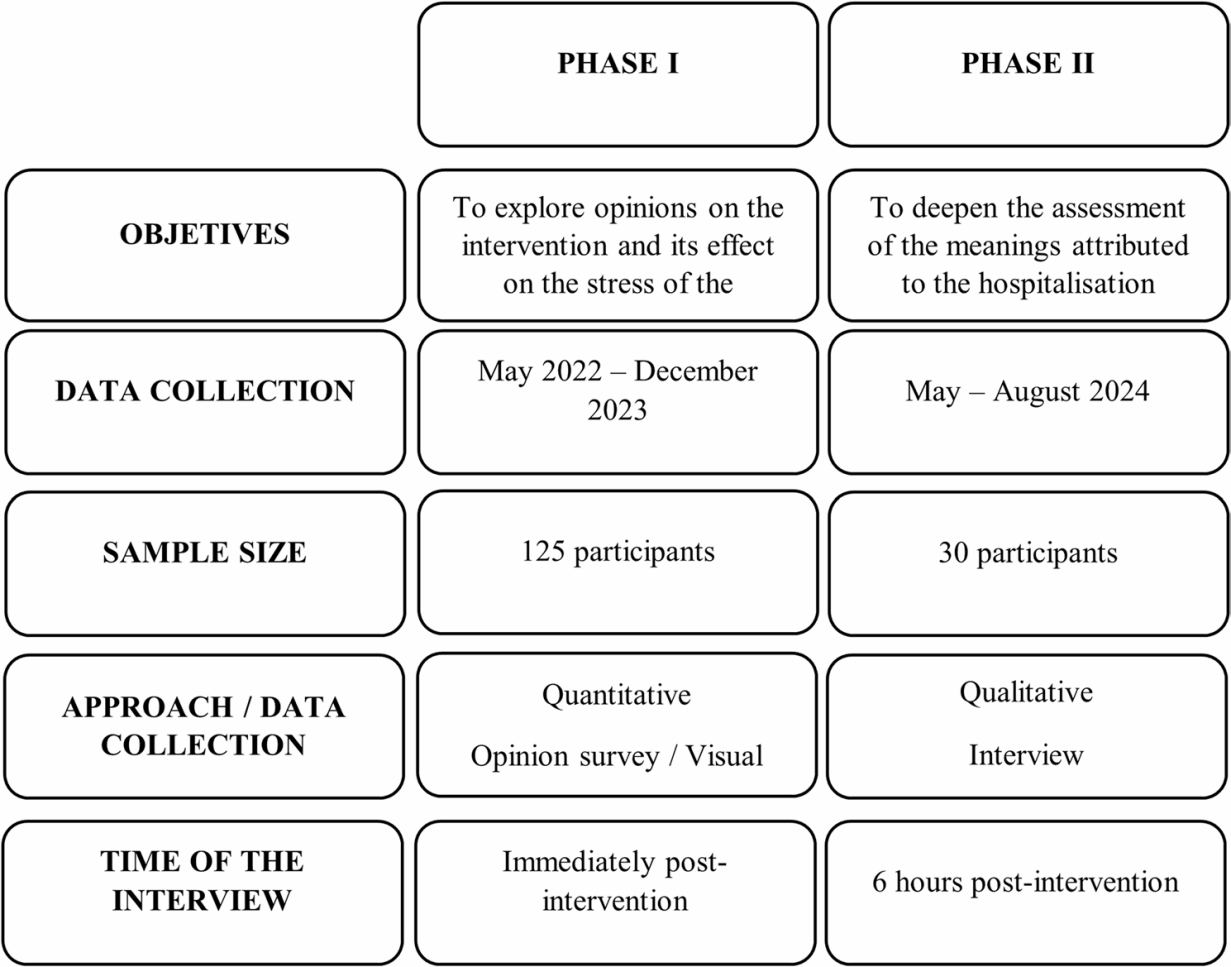
Characteristics of study phases

The research was conducted in the paediatric ward of a teaching hospital affiliated with the School of Medicine, University of São Paulo, Brazil’s leading medical school. It was a quasi-experimental, cross-sectional study with a mixed-methods approach, carried out in two phases (Fig. 1). The study adhered to ethical principles and was reviewed and approved by the institutional ethics committee under registration CAEE 5.804.585.

### Characterization of the intervention

String instruments - kantele for children under 9 years and lyre for those older - were played for 20 to 40 min, depending on the child’s age and receptiveness. Some authors suggest that live harp, small harp (kantele), and lyre music offer greater therapeutic benefits than other musical approaches, due to their pentatonic construction, which naturally resonates with the human body and promotes relaxation and emotional balance. Although historically used for therapeutic purposes, these instruments remain underexplored in studies involving hospitalised children [17]. The intervention also incorporated singing therapy and/or spoken art, tailored to the child’s age. For children under 3 years, phonemes and lullabies in fifth intervals were used; for those over 3 years, poems, storytelling, fables, and mythological tales were included. All interventions were delivered by the same performer.

### Phase 1- quasi-experimental approach

Exploratory phase examined the effects of the intervention on stress in children and caregivers. Sample size was calculated based on a pilot study with data from the first 20 participants. Considering pilot study results—pre-intervention perceived stress mean score of 6.05 (SD 2.59) and post-intervention mean of 5.4 (SD 2.25) and a 5% alpha error, 80% power, and an additional 15% to account for potential attrition, the estimated sample size was 124 participants.

A convenience sampling approach, a non-probabilistic method based on accessibility at the time of data collection, was used. Owing to the nature of the intervention, blinding was not possible. Recruitment was based on clinical stability, the ability of the patient to interact and respond to the intervention, and expected length of stay.

Inclusion criteria comprised patients aged 0 to 14 years who were clinically stable, without severe neuropathy, not receiving sedative medication, and with no planned discharge or transfer on the day of the intervention. Exclusion criteria were children and adolescents in isolation due to contact and/or airborne precautions, or those who were transferred to undergo scheduled examinations during data collection. After identifying eligible patients and obtaining written informed consent from legal guardians, the intervention was administered.

The researcher administered a questionnaire (Appendix 1), developed by the research team, prior to the intervention to characterise the sample and assess perceived stress in children/adolescents and caregivers. The scores for both groups were based on caregiver proxy reports. Immediately after the intervention, participants were again asked about perceived stress and their opinion of the intervention. Stress was graded using a visual analogue scale (VAS), like that used in pain assessments, scoring stress from 0 to 10. Comparison of pre- and post-intervention perceived stress scores was conducted using the Wilcoxon test, as the distribution was non-normal according to the Shapiro–Wilk test.

### Phase 2 – qualitative approach

Qualitative phase explored the meanings attributed to the hospitalisation experience before and after the intervention. Using a convenience sample, this stage included caregivers over 18 years old who were present before, during, and remained with the child for six hours following the intervention. Those who were not in the ward at the time of data collection—due to personal matters, transfer, or the child’s discharge—were excluded. The participants in this phase were not the same as those in Phase I, as this stage took place after the quantitative phase to complement the initial findings. Although the participants were different, the composition of the sample did not present significant differences between the two phases of the study, which does not compromise the generalisability of the results.

Data were collected through semi-structured interviews guided by a script of open-ended questions developed by the researchers, who have extensive experience in qualitative studies (Appendix 2). To minimise response bias, interviews were conducted by a different researcher from the one who delivered the intervention. This researcher, a nurse, received both theoretical and practical training from a university professor with expertise in qualitative research. The theoretical training involved clarification of the foundations of qualitative research and how it should be conducted in clinical practice. Subsequently, the nurse and the professor entered the field together to initiate data collection, engaging in debriefing discussions after each session to enhance data extraction. After five interviews, the nurse proceeded to conduct interviews independently, with the professor reviewing the material and offering suggestions for improvement at the end of each interview.

The interviews were conducted six hours after the intervention, at the bedside. In order to ensure greater privacy for the caregivers, the researcher informed the healthcare team in advance so that the data collection would not be interrupted. All interviews were audio-recorded using two electronic devices and fully transcribed by a separate researcher. A double-checking process was carried out after transcription to ensure accuracy. All transcripts were read by three researchers, who jointly assessed data saturation. Theoretical saturation was determined by consensus, based on the premise that additional interviews would not yield new relevant information [18], and was considered after 30 interviews.

The data were analysed using two approaches. First, a thematic content analysis was conducted following Bardin’s framework [19]. This involved an initial skimming of the material, with each interview read between five and ten times. After becoming familiar with the content, data extraction began, focusing on the identification of potential themes. This analysis was carried out independently by two researchers, followed by discussions to reach a consensus on the final interpretation.

Second approach complemented the first by employing the IRAMUTEQ^®^ software (Interface de R pour les Analyses Multidimensionnelles de Textes et de Questionnaires) [20] to conduct a lexical analysis. This software was selected for its capacity to perform statistical lexical analyses on qualitative data, based on word frequency and associations. Descending Hierarchical Classification (DHC) was used to identify the most frequent words in the narratives and to group them into themes—aligned with Bardin’s thematic analysis—through a chi-square test, with *p* < 0.05 considered statistically significant. In addition, the similarity tree was used to visually represent the semantic map of word connections divided by the identified themes.

## Results

The first phase of data collection occurred between May 2022 and December 2023, involving 125 participants [Appendix 3]. The children had a mean age of 30.6 months and were hospitalised primarily due to respiratory conditions (74.4%). The caregivers interviewed were mostly mothers (80.8%), with a mean age of 29.8 years (Table 1).

**Table 1:** Characteristics of children/adolescents and caregivers – Phase I (*N* = 125)

Characteristics of Children/Adolescents	
	Min – Max (mean; median)
Age (months)	1–172 (30.6; 12)
Length of hospital stay (days)	1–145 (8.0; 4)
	N** (%)**
Gender	
Male	64 (51.2)
Female	61 (48.8)
Race	
White	68 (54.5)
Others	57 (45.6)
Main diagnosis	
Respiratory condition	93 (74.4)
Orthopaedic problem	9 (7.2)
Dermatological problem	4 (3.2)
Post-operative	4 (3.2)
Others	15 (12.0)
Characteristics of Caregivers	
	Min – Max (mean; median)
Age (years)	18–62 (29.8; 27)
	N (%)
Relationship to the patient	
Mother	101 (80.8)
Others	24 (19.2)
Education level	
Primary education	37 (29.6)
Secondary education	70 (56.0)
Higher education	18 (14.4)

**Fig. 2:**
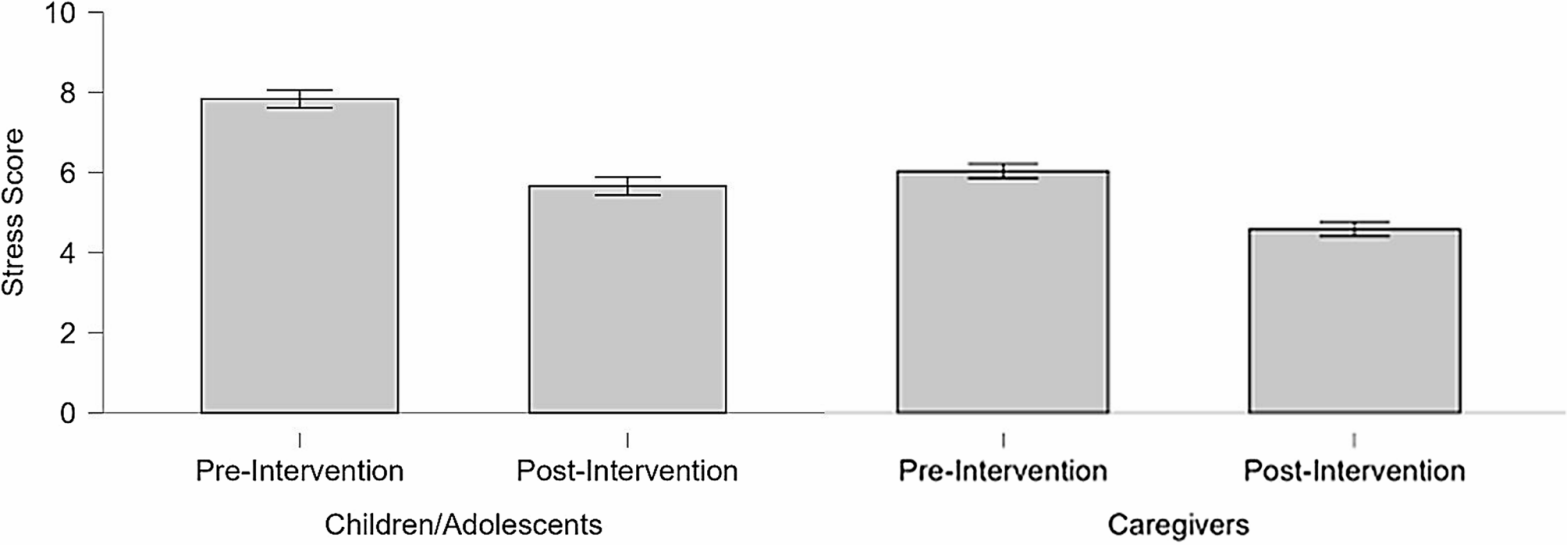
Variations in stress scores of children/adolescents and caregivers, pre- and post-intervention – phase I (*N* = 125)

Comparison of perceived stress scores before and after the intervention demonstrated a significant reduction for both children/adolescents and caregivers (*p* < 0.001), with a large effect size (Cohen’s d = 1.0) (Table 2; Fig. 2). In this initial phase, 100% of participants approved of the intervention, and 74.4% noted behavioural changes in the child or adolescent.

**Table 2:** Stress scores of children/adolescents and caregivers, pre- and post-intervention – phase I (*N* = 125)

	Stress Score Pre-Intervention Mean (95% CI)	Stress Score Post-Intervention Mean (95%CI)	W	*p*-value*	Effect size
Children/Adolescents	7.84 (7.32–8.36)	5.66 (5.15–6.16)	2.556	< 0.001	1.0
Caregivers	6.04 (5.33–6.75)	4.59 (4.02–5.15)	3.240	< 0.001	1.0

* Wilcoxon test

The second phase of the study was conducted between May and August 2024. Of 38 caregivers invited for an interview, 30 participated, and no further interviews were needed after theoretical saturation was reached [Appendix 3], totalling 170 min and 20 s of recordings. Lexical analysis of the interviews generated 164 text segments, of which 84.7% were considered satisfactory, resulting in 5089 words, forms, or vocabularies.

**Fig. 3:**
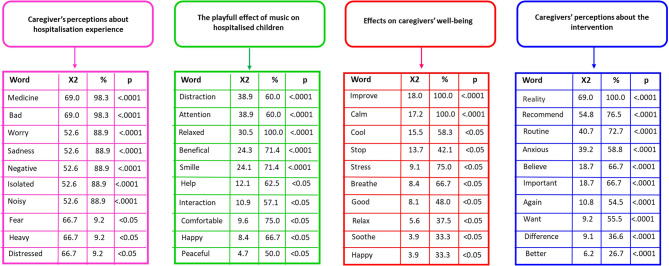
Categories of descending hierarchical classification – phase II (N = 30) Note: The DHC presents the most frequent words (%) within each of the identified themes, represented by different colours. Words were grouped based on their lexical similarities, reflecting shared meanings in the formulation of the discussed content. Through the chi-square test (χ²), it was possible to identify the terms with the highest frequency and statistical significance (p), contributing to the construction and delimitation of each thematic class

From the analysis, four themes emerged (Fig. 3). In summary, hospitalisation was perceived by caregivers as a challenging context that negatively impacted both their well-being and that of the child. However, following the intervention, caregivers described it as a moment of relief within a stressful environment, with mutual benefits for both parties, and perceived the intervention positively.

In the first category, *Caregivers’ perception of the hospitalisation experience*, the vast majority of caregivers described hospitalisation as negative (*p* < 0.001), bad (*p* < 0.001), and burdensome (*p* < 0.05). It was associated with feelings of worry (*p* < 0.001), fear (*p* < 0.05), distress (*p* < 0.05), and sadness (*p* < 0.001), as well as with characteristics of the hospital environment such as noise (*p* < 0.001), isolation (*p* < 0.001), and the presence of medications and procedures (*p* < 0.001).

The following interview excerpts illustrate these findings:

*“When they came to draw her blood… she already has trauma*,* she sees someone in white and she knows. It’s very hard to find her vein*,* and she keeps crying.*” (C3).

”*We’re in the middle of a whirlwind — people coming and going*,* turning the child this way and that… We’re stuck in this routine of nurses*,* doctors*,* and medications*.” (C5).

In the second category: *the playful effect of music on hospitalised children*, caregivers described the intervention as a playful moment that helped (*p* < 0.05) the child and promoted benefits (*p* < 0.001). They reported that the intervention provided relaxation (*p* < 0.001), comfort (*p* < 0.05), and tranquillity (*p* < 0.05). After the intervention, caregivers observed that the children appeared happier (*p* < 0.05) and smiled more (*p* < 0.001).

*“My son became very calm with the music. He smiled immediately*,* paid attention*,* kept looking and laughing at her. He became really peaceful… While she was here*,* my son kept watching her*,* laughing*,* very calm. She left*,* and he practically fell asleep. Before*,* he was very agitated*,* but after the music*,* and even now*,* he has remained very calm. Everyone who comes in now—he no longer cries*,* he stays calm and lets them handle him.” (C2)*.

In the third category, *Effects on caregivers’ well-being*, caregivers reported that the intervention promotes calmness (*p* < 0.05) and relaxation (*p* < 0.05), and that they felt happier (*p* < 0.05) after it ended. They expressed a sense of being able to pause (*p* < 0.05) and breathe (*p* < 0.05) amidst an exhausting routine marked by challenges. Only one caregiver felt that the intervention did not help and expressed unwillingness to hear the music again. Possible reasons for this result will be addressed in the discussion. The others reported that the intervention brought greater tranquillity (*p* < 0.001) and reduced stress (*p* < 0.05) (Fig. 3), as stated by this caregiver:

“*At the hospital*,* we mothers need support. So when someone comes and does this with music*,* it’s good… Music makes the environment calmer and more peaceful. I was a bit tense and worried — not anymore. I’m calm and confident now*,* after the music and the conversation with her. It was very emotional… I was feeling isolated*,* but after the music*,* I felt much better*.” (C12).

Finally, in the last category, *Caregivers’ perceptions of the intervention*, caregivers stated that they would recommend (*p* < 0.001) the intervention to other children and caregivers. It was perceived as important (*p* < 0.001), something that should be incorporated into the institutional routine for making a difference (*p* < 0.001), and they expressed a desire to receive it again (*p* < 0.001) in the future (Fig. 3):

“*It would be wonderful to have this more often — I would recommend it to other parents and family members. It would be great if it were implemented*,* if it became a standard in hospitals*,* with this intervention happening at least once a day*.” (C11).

**Fig. 4:**
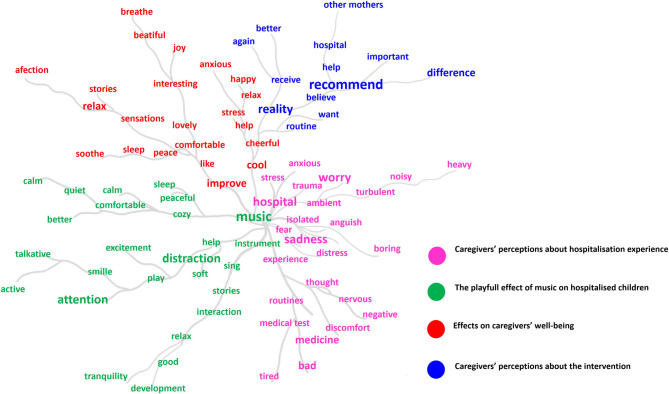
Similarity tree – Phase II (N = 30) Note: The similitude tree illustrates the words that appeared most frequently in each theme, separated by colours, allowing the identification of their connections within the participants’ narratives. Each line drawn represents the integration and relationship between terms, highlighting how these elements were interconnected in the construction of the discourse

Figure 4 illustrates the connection between words within the participants’ narratives that supported the identified themes.

The main excerpts from the interviews, along with the respective caregiver identification (C), will be presented in the discussion section.

## Discussion

Various CIHP have been implemented to humanised hospital care. The present study assessed the effects of an MBI and identified that the intervention yielded benefits for hospitalised children/adolescents and their caregivers, corroborating previous literature findings.

In general, participants described the hospital environment as uncomfortable and procedure-centred, as exemplified in the following interview excerpts:

*‘The hospital is a tense place… It’s chaos for us in hospital.’ (C13)*.

*‘My niece was already stressed; three doctors had come to check her*,* and then physiotherapy… The hospital is so overwhelming*,* people just come in to manipulate the child*,* to poke*,* to suction…’ (C23)*.

Other authors have reported similar perceptions of hospitalisation, regardless of the cultural and socioeconomic characteristics of the populations studied. A study conducted in the Pacific Northwest region of the United States found that parents of hospitalised children reported physiological challenges (sleep, eating, and personal hygiene difficulties), emotional distress (feelings of isolation and stress), and communication challenges with the healthcare team [21]. Importantly, negative perceptions were associated less with the severity of diagnosis than with environmental and care-related factors, underscoring the value of humanised care and strong team–family relationships. Such practices promote inclusive hospital spaces, alleviate suffering, and help families reframe the hospitalisation experience [6, 22], reinforcing the relevance of this study’s findings.

The reduction in stress scores for both paediatric patients and their caregivers may be attributed to the emotional and physiological modulation induced by sound [11, 23]. Music helps individuals express feelings, which is particularly important among children and adolescents, who often struggle to verbalise fears, worries, and anxiety. Sampaio (2023) described different experiences provided to children and adolescents with chronic illnesses, observing positive effects on psycho-emotional outcomes and noting that music stimulates playfulness and promotes children’s agency [24]. This was echoed in caregivers’ comments:

*‘He paid close attention while she played and sang*,* focusing intently on the instruments*,* very interested. He stayed calm the whole time. At first*,* I thought he would cry*,* but no—he laughed and was cheerful.’ (C6)*.

*‘My daughter paid a lot of attention*,* really enjoyed it. She loves stories*,* so she was excited. Today she’s different from other days—walked more*,* played more*,* was calmer*,* less anxious*,* less fearful*,* and slept better.’ (C8)*.

*‘It captured the attention of all the children; they stayed very quiet*,* listening to the story… My son even stopped playing with his mobile phone*,* focused on the story and music*,* calm*,* almost falling asleep.’ (C11)*.

Music combined with movement, as in this intervention, stimulates multiple sensory and neural pathways, supporting interaction, cognition, communication, and motor skills. It also activates the prefrontal cortex, enhancing self-regulation and attention, and is linked to the release of endorphins, dopamine, and other neurotransmitters that promote well-being, reduce suffering, and lessen the need for sedative or analgesic drugs [14, 23, 25–28]. These mechanisms may account for the study’s findings, both in lower stress scores and in the experiences reported in interviews:

*‘The intervention was very peaceful; it helped him. The environment became calm; my son was quiet. It was soft music; he even looked relaxed*,* paying attention.’ (C4)*.

*‘She played while my son was sleeping*,* and he became even more relaxed. I thought it was great—even the children nearby relaxed along with him… The music is very soothing; it relaxes and improves the child.’ (C7)*.

Similar effects were observed among caregivers:

*‘I think the music calms the child and also the parents. I felt myself relaxing—she came*,* sang*,* and I felt calm. Both my son and I fell asleep afterwards. I felt good*,* calm*,* and relaxed.’ (C2)*.

*‘I think it was a moment of peace and tranquillity. I managed to rest*,* to have peace during that moment… It brought benefits for the child but also for me (mother).’ (C5)*.

*‘I love music. I know it brings benefits*,* calm*,* and peace*,* so I really liked it. I felt better after she came to play. It calmed me; the sound of the instrument was beautiful… She brought serenity*,* a peace I found important. I felt better and comforted by the music—I really liked it.’ (C20)*.

It is worth noting that stress and anxiety reduction in caregivers may indirectly yield positive effects for children/adolescents. This appears particularly relevant for younger children, for whom the bond with caregivers is critical to well-being and a sense of security [29]. Studies of musical interventions in neonatal units indicate that maternal anxiety reduction mediates well-being in premature infants [17]. These findings suggest that music therapy may work bidirectionally, benefiting both children and families by fostering a calmer, more supportive environment. These bidirectional effects highlight the importance of family-centred care, which recognises the interconnected emotional wellbeing of both child and caregiver.

The intervention was highly accepted, and participants expressed a desire for it to become a routine hospital practice:

*‘I believe that if music were implemented in hospitals*,* it would be very interesting and would cheer up the children*,* helping them forget what they’re going through in hospital… I’d recommend it to other mothers.’ (C6)*.

*‘I’d like to have it again*,* whenever necessary*,* whenever possible. It would be worthwhile having this in hospitals; it would be good for hospitalised children and adults too. It would be worth investing in.’ (C9)*.

However, it is important to note that CIHP achieve better outcomes when they respect individual needs, preferences, beliefs, and values [30, 31]. A patient- and family-centred approach is considered essential for successful CIHP implementation in care [9]. The importance of respecting individual preferences was illustrated by the one caregiver who did not approve of the intervention:

*‘Although the music was good*,* I felt palpitations and anxiety; I didn’t feel calm… The music was tedious*,* too soft—it’s not the type of music my daughter likes.’ (C18)*.

Regarding music, cultural adaptation is crucial. Music is more than just an auditory stimulus - it carries different meanings for each person, depending on their biography and sociocultural context. Choosing musical repertoires aligned with patients’ cultural preferences can enhance therapeutic effects, promoting greater engagement, comfort, and familiarity. Therefore, interventions should include varied instruments and repertoires to accommodate diverse tastes.

This study has limitations. First, the sample was one of convenience and, although the size was adequate for the proposed objectives, its homogeneous composition, particularly regarding sociocultural aspects, and limits generalisability. Notably, the absence of a control group restricts causal inferences, and the reliance on caregiver opinions and interviews, without perspectives from children/adolescents or other professionals, further limits the findings. On the other hand, the sample was not restricted to a specific condition, instead encompassing patients from a general paediatric ward. The study design took into account that children remain hospitalised in the ward for an average of 3 to 4 days, making it unfeasible to conduct a longitudinal study to assess the persistence of the observed effects. The study relied on subjective data and did not include objective methods for evaluating intervention effects. Nevertheless, the qualitative approach allowed for the identification of intervention acceptability and provided insights into its impact on both paediatric patients and their caregivers. The discussion addressed various cultural aspects and enhanced the practical implications, potentially contributing to the field of paediatric humanised care.

One aspect to be considered is the limited number of qualified professionals available to deliver MBIs in hospital settings, particularly in low- and middle-income countries. Strategies to overcome these challenges may include volunteer programmes and partnerships established with music schools.

Future research should explore different art and music modalities, using varied instruments and repertoires, as well as studies involving digital or recorded music, which are more scalable alternatives. Additionally, studies employing diverse methodological approaches, such as assessments based on physiological parameters, validated scales, and stress biomarkers, will be valuable. Including the perspectives of child/adolescent, with age-appropriate methods, and healthcare teams would further broaden understanding of the effects and feasibility of CIHP in different clinical contexts.

In conclusion, this study adds evidence supporting the benefits of MBIs for hospitalised children, adolescents, and their families, offering insights for incorporating such practices into paediatric care. The results reinforce the role of CIHP, particularly through art and music therapy, as effective strategies for humanised hospital care. These findings suggest that incorporating music-based practices may significantly contribute to creating more welcoming, interactive hospital environments that are responsive to the emotional and psychological needs of patients and their families.

## Supplementary Information

Supplementary Material 1.

Supplementary Material 2.

Supplementary Material 3.

## Data Availability

No datasets were generated or analysed during the current study.

## Abbreviations

AAP: American Academy of Pediatrics
CAEE: Certificate of Presentation for Ethical Appreciation
CIHP: Complementary and Integrative Health Practices
DHC: Descending Hierarchical Classification
IRAMUTEQ® software: Interface de R pour les Analyses Multidimensionnelles de Textes et de Questionnaires
MBI: Music-Based Intervention
VAS: Visual Analogue Scale
WHO: World Health Organization
