# Supplementary material for: The healing power of music: a mixed-methods study on stress reduction in paediatric hospitalisation
Source: BMC Complement Med Ther. 2025 Oct 17;25:386. doi: 10.1186/s12906-025-05098-0 (PMC12535118; doi:10.1186/s12906-025-05098-0)
Supplement: Supplementary file 1 — Supplementary Material 1. [file 12906_2025_5098_MOESM1_ESM.docx]

**Questionnaire – Phase I**

| Date of interview |  |
| --- | --- |
| Identification number |  |
| Name of child/adolescent |  |
| Date of birth |  |
| Race (as reported by the caregiver) |  |
| Date of hospitalisation |  |
| Main diagnosis |  |
| Name of the caregiver |  |
| Caregiver’s date of bith |  |
| Relationship to patient | ☐ mother ☐ father ☐ other |
| Education level | ☐ primary ☐ secondary ☐ higher |

**Considering the following stress score scale, in which 0 corresponds to “no stress” and 10 corresponds to the “highest level of stress”, what score would you assign to the child’s/adolescent’s stress related to hospitalisation? And to your own level of stress?**


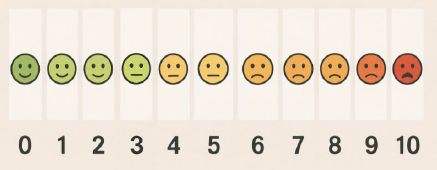


| **Perceived stress pre-intervention** | **Score** |
| --- | --- |
| Child/adolescent |  |
| Caregiver |  |
| **Perceived stress post-intervention** |  |
| Child/adolescent |  |
| Caregiver |  |

| Did you notice any changes in the childs/adolescent’s behaviour after the musical intervention? | ☐ yes ☐ no |
| --- | --- |
| Did you like the musical intervention? | ☐ yes ☐ no |
