# Supplementary material for: The healing power of music: a mixed-methods study on stress reduction in paediatric hospitalisation
Source: BMC Complement Med Ther. 2025 Oct 17;25:386. doi: 10.1186/s12906-025-05098-0 (PMC12535118; doi:10.1186/s12906-025-05098-0)
Supplement: Supplementary file 2 — Supplementary Material 2. [file 12906_2025_5098_MOESM2_ESM.docx]

**Guiding Questions for the Qualitative Interview – Phase II**

1. What do you think about the hospital environment? What does hospitalisation represent to you?
2. Now, could you tell me a bit about how the musical intervention took place?
3. What did you think about the musical intervention?
4. Could you describe how you felt during the musical intervention?
5. And how did you feel after the intervention was over? Why?
6. And the child/adolescent – how did they behave during the musical intervention?
7. How did he/she feel after the intervention? Why?
8. Do you believe the intervention may have brought any benefits to you? If so, which ones? Why?
9. And to the child/adolescent - Do you believe the intervention may have brought any benefits to he/she? If so, which ones? Why?
10. Would you like to receive the musical intervention again? Why?
11. Would you like this intervention to become part of the hospital’s routine care? Why?
