# Supplementary figures and images for: The healing power of music: a mixed-methods study on stress reduction in paediatric hospitalisation
Source: BMC Complement Med Ther. 2025 Oct 17;25:386. doi: 10.1186/s12906-025-05098-0 (PMC12535118; doi:10.1186/s12906-025-05098-0)

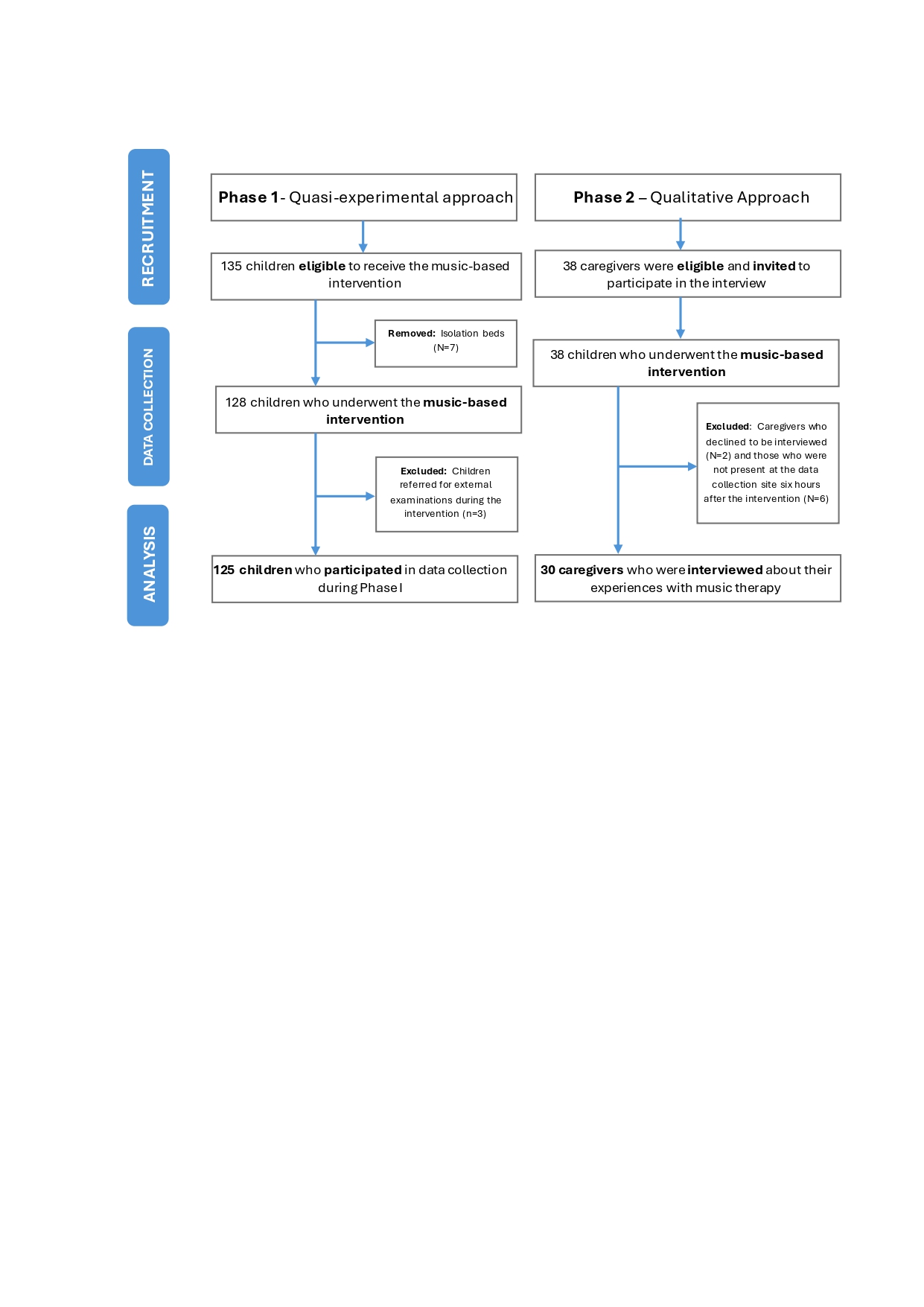

Supplement: Supplementary file 3 — Supplementary Material 3. [file 12906_2025_5098_MOESM3_ESM.jpg]
